# Impressions of sexual unfaithfulness and their accuracy show a degree of universality

**DOI:** 10.1371/journal.pone.0205716

**Published:** 2018-10-25

**Authors:** Clare A. M. Sutherland, Laura M. Martin, Nadine Kloth, Leigh W. Simmons, Yong Zhi Foo, Gillian Rhodes

**Affiliations:** 1 ARC Centre of Excellence in Cognition and its Disorders, School of Psychological Science, University of Western Australia, Crawley, WA, Australia; 2 Centre for Evolutionary Biology and School of Biological Sciences, University of Western Australia, Crawley, WA, Australia; Universita degli Studi di Udine, ITALY

## Abstract

Forming accurate impressions of others’ trustworthiness is a critical social skill, with faithfulness representing a key aspect of trust in sexual relationships. Interestingly, there is evidence for a small degree of accuracy in facial impressions of sexual unfaithfulness. Theoretical accounts suggest that these impressions may function to help with partner selection, and may be universal. If so, impressions should be similar for perceivers from different cultures and accuracy should not be limited to own-race faces. We tested these predictions by asking Caucasian and Asian women to judge the likelihood of unfaithfulness from the faces of Caucasian males whose past sexual history was known. In two studies we found high cross-cultural agreement in these impressions, consistent with universality in the impressions themselves. In Study 1, we found an other-race effect in impression accuracy, with significantly less accurate cross-race impressions by Asian women than own-race impressions by Caucasian women. Asian women showed no accuracy. Interestingly, in Study 2, Asian women who had grown up in the West showed small but significant accuracy in their impressions, with no other-race effect. Results are consistent with a degree of universality in the accuracy of this important aspect of social perception, provided that perceivers have experience with the faces being assessed.

## Introduction

Forming first impressions of others’ trustworthiness is a critical aspect of human social perception [1,2]. People readily form facial impressions of trustworthiness from photographs of unfamiliar strangers, are able to make these judgments from a split second exposure to a face, and, to some extent, agree on which faces look trustworthy (see [3] for a review). Trustworthiness impressions are suggested to be especially critical for social perception because they reflect the by-products of adaptive mechanisms for judging threat [1,2]. This explanation in turn suggests that the tendency to judge others based on their facial trustworthiness is perhaps a universal aspect of social cognition. In support, facial impressions of trustworthiness and related traits show considerable cross-cultural agreement, with perceivers from different cultures forming similar impressions of the same faces [4–7].

A crucial aspect of trustworthiness in the context of romantic relationships is sexual unfaithfulness [8]. Intriguingly, there is very small but above-chance accuracy for judgments of the likelihood of sexual unfaithfulness made to opposite sex faces [9,10]. Accuracy is adaptive, because pairing with a partner who engages in extra-pair sex could confer substantial fitness costs [9,10]. Thus, facial unfaithfulness impressions may help us assess threats to evolutionary fitness. Unfaithfulness impressions therefore have a potential functional significance, and might also be expected to be universal.

If facial impressions of unfaithfulness are universal, then there should be both agreement and above-chance accuracy across perceivers from different cultures when forming these impressions from the same faces. Although there is good evidence for high agreement across perceivers from different cultures for many facial impressions [6], facial impressions of unfaithfulness have yet to be tested. Moreover, cross-cultural agreement may not necessarily translate into cross-cultural accuracy. Little is known about how accurate facial impressions are across culture.

A functional account of unfaithfulness impressions would predict that these judgments have some accuracy, given that infidelity will carry fitness costs across cultural contexts. This argument has also been made for cross-cultural accuracy in facial impressions of sexual orientation, another critical judgment in the formation of sexual relationships [11]. Indeed, perceivers are modestly accurate in judging sexual orientation across perceiver culture, for both own-race and other-race faces [11] (Note that we use the term ‘race’ to refer to visually distinct socio-cultural groups, not biological categories [12]). Similarly, we would expect some cross-cultural accuracy in unfaithfulness impressions. We note that although accuracy here is very small, even a small degree of accuracy could be selected for, given its likely consequences for reproductive success [13]. Indeed, any degree of accuracy from facial impressions would be expected to be very small, as observed, because targets may in turn have undergone selection pressure to mask signals of unfaithfulness [14].

Finally, any accuracy may also depend on a match between perceiver and face race. A large literature has found that people are more accurate with own-race than other-race faces on a variety of face perception tasks [15,16]. These *other-race* effects are widespread, with reduced accuracy for other-race faces when judging facial identity [17,18], emotional expression [19,20], gaze direction [21] and even occupational success ([22]; but see [23]). Reduced perceptual experience with other-race faces could well reduce any sensitivity to facial cues that validly signal unfaithfulness [16]. Therefore, an other-race effect would likely also be found for accuracy in sexual unfaithfulness judgments. Regardless of an other-race effect, any accuracy in judging other-race faces would be consistent with universality.

### Current studies

Here we tested the agreement and accuracy of these impressions across culture for the first time. We examined Caucasian and East Asian women’s impressions of the likely unfaithfulness of Caucasian men. We focused on women’s impressions only, as previous research has shown above-chance accuracy for women judging male faces but not the reverse (at least with ratings: [9,10]) and we examined impressions of opposite-sex faces, as these are most relevant to mate choice. The women judged Caucasian male faces for which self-reported sexual unfaithfulness data were available [10,24]. We know of no database of Asian faces with associated sexual histories and so were unable to implement a fully crossed design with both Asian and Caucasian faces. Nevertheless, above-chance accuracy in Asian women’s impressions of the Caucasian male faces would support a degree of universality in accuracy of unfaithfulness impressions. Good agreement in the impressions of the two groups of perceivers would support universality in the impressions themselves.

In Study 1, we examined the agreement and accuracy of sexual unfaithfulness judgments of the Caucasian male faces by Caucasian and Asian women who had grown up in predominantly Caucasian or Asian countries, respectively. We expected to find high agreement in unfaithfulness impressions across perceiver race, based on a similar pattern for other trait judgments [6]. We also expected to find above-chance accuracy for Caucasian impressions, replicating previous studies [9,10]. Given the ubiquitous other-race effects in face perception, we anticipated reduced accuracy in the Asian women’s impressions, because these were for other-race faces [16,18]. Nevertheless, Asian women could still show above-chance accuracy. In Study 2, we tested whether Asian women with more extensive experience with Caucasian faces would show any accuracy, by recruiting new Asian women who had grown up in the West. Study 2 also included a direct replication for the Caucasian women. Above-chance accuracy for either Asian group would be consistent with a degree of universality and an adaptive role for impressions of likely unfaithfulness.

Across studies, we examined potential mediators of any accuracy in unfaithfulness judgments, chosen based on previous studies. These included perceptions of attractiveness, as people may infer that more attractive individuals will have more opportunity to be unfaithful, and perceptions of masculinity, which is a valid cue to unfaithfulness in men [10,25]. We also included untrustworthiness judgments, to confirm that accuracy is specific to unfaithfulness, as found in previous studies [9,10], rather than reflecting more general impressions of untrustworthiness. In Study 1 we additionally confirmed that each group had less experience with other-race than own-race faces, and showed the expected other-race effect in facial recognition [15,16].

## Study 1

### Method

#### Participants

Thirty-two East Asian (Chinese) and 50 Caucasian adult females from the University of Western Australia community participated for course credit (*n* = 53), a $10 honorarium (*n* = 18) or as volunteers (*n* = 11). Participants provided written informed consent and ethical approval was provided by the Human Research Ethics Board of the University of Western Australia. Sample size was based on a previous study [10], so that we tested all women who signed up during the pre-specified testing period (July-September 2015), with a minimum of 30 participants per group. The Caucasian women had spent the majority of their lives in Australia or another predominantly Caucasian country (M = 18.7 years, SD = 5.1 years). The Asian women had not lived in Australia or any other predominantly Caucasian country for more than 36 months (*M* = 13 months, *SD* = 11 months). We excluded five additional Caucasian and nine Asian women (who had previously seen the faces; lived in Asia or Australia for more than 36 months, or were homosexual), before any analyses. Categorization was self-reported.

The Asian women had a mean age of 20.7 years (*SD* = 1.3, range 19 to 23). To be conservative, we age-matched the Caucasian group (mean = 19.9 years, *SD* = 3.0, range = 17 to 32) with the Asian group, so that age-related increases in face perception abilities [26] could not explain any difference between the groups (no group difference: *t*(79.96) = 1.64, *p* = .106, *d* = 0.35). To age-match the groups, we excluded an additional eight Caucasian participants above 32 years old (1.5x the interquartile range). Including these did not change the results.

We confirmed that our participant groups had more social contact with own-race than other-race individuals, using a pre-existing questionnaire [27]. Caucasian women reported significantly more social contact with Caucasian (M = 5.4, SD = 0.6) than Asian (M = 2.7, SD *=* 1.0) individuals: *t*(49) = 14.86, *p* < .001, *d* = 3.27. Conversely, the Asian women reported significantly more social contact with Asian (M = 5.0, SD = 0.6) than Caucasian (M = 2.9, SD = 0.6) individuals: *t*(31) = 12.07, *p* < .001, *d* = 3.50.

#### Stimuli

Front-view, color photographs (420 pixels in height) of 100 Caucasian adult male faces with neutral expressions were taken from a pre-existing database [24], excluding one face that was potentially familiar to the current participants and one with unfeasibly high sexual infidelity scores (see [10] for details). An oval mask hid the hair, but left the face contour and inner hairline visible. Two additional faces were used for practice.

Target infidelity was measured using the infidelity index taken from a previous study [10]. This index consisted of scores derived from a single principal component, which combined self-reported cheating (number of extra-pair copulation partners) and poaching (number of sexual partners already in another relationship) [10]. The original reporting conditions were carefully designed to encourage honesty: participants responded anonymously and in isolation, and lodged their answers in a locked box [24].

#### Procedure

Participants first rated the 100 faces on their unfaithfulness, defined as infidelity in a sexual relationship. On each trial, a face appeared for three seconds, followed by the question, ‘How likely is this person to be unfaithful?’ with a 10-point scale shown underneath (*1 not very likely*, *10 extremely likely*). Ratings were made using labeled keyboard keys and participants initiated each trial by pressing the spacebar. Participants were informed that there were no right or wrong answers, and encouraged to use the entire scale.

Next, participants were randomly assigned to rate these same faces either on attractiveness (*n* = 17 Caucasian, *n* = 10 Asian), masculinity (*n* = 16 Caucasian, *n* = 13 Asian) or untrustworthiness (*n* = 17 Caucasian, *n* = 9 Asian). This procedure was identical to the sexual unfaithfulness ratings, except that participants instead rated the faces from 1 (*not at all*) to 10 (*extremely*) attractive, masculine or untrustworthy. Unfaithfulness was always rated first to avoid carryover effects from the other ratings [28]. Faces appeared in random order.

Participants then completed the Australian and Chinese Cambridge Face Memory Tests (CFMT) [29,30], in counterbalanced order. Finally, participants reported their social contact with Caucasian and Asian individuals (using a validated questionnaire [27]), their ethnicity, sexual orientation and time spent abroad to establish whether they met our demographic criteria. The experiment took about 45 minutes.

### Results and discussion

Judgments of unfaithfulness showed good reliability at the group level (Cronbach’s alpha for Caucasians: 0.91; Asian: 0.81), as did attractiveness (Caucasians: 0.92; Asian: 0.77), masculinity (Caucasians: 0.91; Asian: 0.81) and untrustworthiness (Caucasians: 0.81, although less good for the Asian ratings: 0.60). We obtained a mean rating for each face on each attribute for Caucasian and Asian perceivers, by averaging ratings of each group separately (**Table 1**). Caucasian attractiveness ratings, Asian unfaithfulness and untrustworthiness ratings and the infidelity index were not normally distributed (Kolmogorov-Smirnov tests < 0.29, *p* < .05, see **Table 1** for skew and kurtosis) so we report non-parametric Kendall’s Tau as well as parametric correlations, although they produce very similar results.

**Table 1:** Descriptive statistics for the age and infidelity of the Caucasian male target faces, and the social judgments by Caucasian and Asian women, Study 1.

		Mean	SD	Range	Skew	Kurtosis
Target faces (*n* = 100)	Infidelity index	0	1.0	-0.5–4.7	3.06	10.49
	Age	24.7	6.9	18–47	1.58	1.84
Caucasian impressions	Unfaithfulness	5.1	0.9	3.0–7.0	0.22	-0.60
	Attractiveness	2.9	1.1	1.1–6.3	0.86	0.31
	Masculinity	5.7	1.2	3.4–9.1	0.23	-0.59
	Untrustworthiness	5.7	1.0	3.5–8.7	0.38	0.08
Asian impressions	Unfaithfulness	5.3	0.7	3.8–7.5	0.42	0.08
	Attractiveness	3.6	1.0	1.5–5.9	0.26	-0.30
	Masculinity	6.0	0.9	3.9–8.3	-0.02	-0.32
	Untrustworthiness	5.9	1.0	3.4–8.9	0.07	0.51

To ensure that our conclusions were robust, we also analyzed the cheating data with generalized linear models with negative binomial distributions (appropriate for count data). These models produced the same conclusions as the main analyses, although some models failed to converge (**S1 Text**).

#### High cross-cultural impression agreement

Impressions of unfaithfulness (and attractiveness, masculinity and untrustworthiness) showed strong agreement between the Caucasian and Asian women, demonstrating considerable cross-cultural similarity (all *r* >.63, all *p* < .001; **Table 2**). The groups showed no significant difference in mean impression levels (all *t*s < 1.44, all *p*s > .163, all *d*s < 0.63; **Table 1**) or in the impression variances (Levene’s tests *F* < 2.71, *p* > .104; **Table 1**), consistent with a high degree of universality in the impressions themselves.

**Table 2:** Relationships amongst impressions of Caucasian male faces made by Caucasian and Asian women in Study 1, and between these impressions and actual infidelity (infidelity index). Relationships measured by Kendall’s tau (above diagonal) and Pearson’s *r* (below diagonal). *P*-values shown underneath.

	Infide. index	Caucasian impressions				Asian impressions			
		Unfaith.	Attract.	Masc.	Untrust.	Unfaith.	Attract.	Masc.	Untrust.
**Infidelity index**	-	.16*	-.03	.14	.04	.07	.00	.08	.03
		.036	.640	.065	.638	.381	.995	.313	.689
**Caucasian impressions**									
Unfaithfulness	.26**	-	.01	.51**	.52**	.50**	-.04	.44**	.29**
	.008		.858	< .001	< .001	< .001	.563	< .001	< .001
Attractiveness	-.06	.01	-	.14*	-.27**	-.05	.64**	.16*	-.21**
	.581	.915		.036	< .001	.424	< .001	.023	.002
Masculinity	.20*	.71**	.27**	-	.20**	.28**	.04	.63**	.12
	.047	< .001	.006		.003	< .001	.559	< .001	.090
Untrustworthiness	.14	.74**	-.43**	.33**	-	.52**	-.32**	.16*	.42**
	.153	< .001	< .001	< .001		< .001	< .001	.020	< .001
**Asian impressions**									
Unfaithfulness	.08	.73**	-.17	.45**	.75**	-	-.18**	.22**	.43**
	.432	< .001	.088	< .001	< .001		.008	.001	< .001
Attractiveness	.01	-.11	.81**	.09	-.51**	-.32**	-	.12	-.23**
	.898	.262	< .001	.371	< .001	.001		.083	< .001
Masculinity	.14	.64**	.30**	.83**	.27**	.36**	.16	-	.08
	.168	< .001	.003	< .001	.006	< .001	.106		.229
Untrustworthiness	.00	.44**	-.38**	.19	.63**	.66**	-.41**	.11	-
	.978	< .001	< .001	.058	< .001	< .001	< .001	.280	

** *p* < .01

*p < .05, all *N* = 100.

We also computed two-way random single measures ICCs to measure agreement amongst individual raters: unfaithfulness (Caucasians .17, CI = .14 - .23, Asian .12, CI = .09 - .16), attractiveness (Caucasians .41, CI = .34 - .49, Asian .26, CI = .19 - .34), masculinity (Caucasians .37, CI = .31 - .46, Asian .24, CI = .18 - .32) and untrustworthiness (Caucasians .20, CI = .15 - .27, Asians .14, CI = .09 - .21). Agreement in judgments tended to be lower for Asian than Caucasian raters and this difference was significant for attractiveness and masculinity (p < .05, based on confidence interval overlap [31]). This finding is perhaps indicative of a subtle own-race bias in agreement in these judgments, set against the overall pattern of high similarity.

#### Accuracy of sexual unfaithfulness impressions

We assessed accuracy in impressions of unfaithfulness by correlating the infidelity index with the average unfaithfulness ratings for each participant group, following [10]. Caucasian (own-race) impressions were significantly above chance, replicating previous findings (*r* = .26, *p* = .008, tau = .16, *p* = .036, *N* = 100, 95% CI: .07 to .43; **Table 2**). As expected, there was an other-race effect, with Asian impressions showing significantly less accuracy, *Z* = 2.47, *p* = .014, *N* = 100 (*Z*-test from [32]), and indeed no accuracy at all (*r* = .08, *p* = .432, tau = .07, *p* = .381, *N* = 100, 95% CI: -.12 to 0.27; **Table 2**). Thus, other-race effects extend to this new domain.

#### Facial cues that mediate accurate unfaithfulness impressions

We examined potential facial cues underlying the accuracy in Caucasian unfaithfulness ratings (Asian ratings were not accurate). Masculinity ratings correlated significantly with both the unfaithfulness ratings and the infidelity index **(Table 2**), making them a potential mediator. Moreover, the partial correlation between the unfaithfulness ratings and the infidelity index was no longer significant with masculinity controlled (partial *r* = .176, *p* = .081, *N* = 100). This pattern replicates Rhodes et al.’s finding that perceived masculinity mediated the accuracy of unfaithfulness impressions [10]. Attractiveness was not a mediator as it was unrelated to either unfaithfulness ratings and actual infidelity (**Table 2**; as [10]). Untrustworthiness ratings were also unrelated to infidelity (**Table 2**), confirming previous findings that accuracy is specific to unfaithfulness impressions [10].

#### Facial cues to unfaithfulness impressions

Finally, we compared the relationships between impressions of unfaithfulness and the other rated attributes separately for the Caucasian and Asian groups, to test whether accuracy differences were due to the groups using different cues to form unfaithfulness impressions. Impressions of unfaithfulness correlated significantly with untrustworthiness and masculinity impressions, for both groups, and negatively with attractiveness for the Asian group only (**Table 2**). However, the relationship with masculinity was stronger for the Caucasian group, *p* < .001 (*Z*-test from [33]). The greater reliance on the valid cue of masculinity by Caucasian women may help explain their increased accuracy over the Asian group.

#### Individual-level accuracy in unfaithfulness impressions

Above, we examined accuracy at the *aggregate* level, by correlating the infidelity index with average unfaithfulness judgments (following [10]). However, aggregation can produce a higher estimate of accuracy than individual participants’ judgments, because it removes noise, errors and participant disagreement [34,35]. Accuracy has not previously been examined at the individual participant level for ratings of sexual unfaithfulness of these faces.

**Fig 1:**
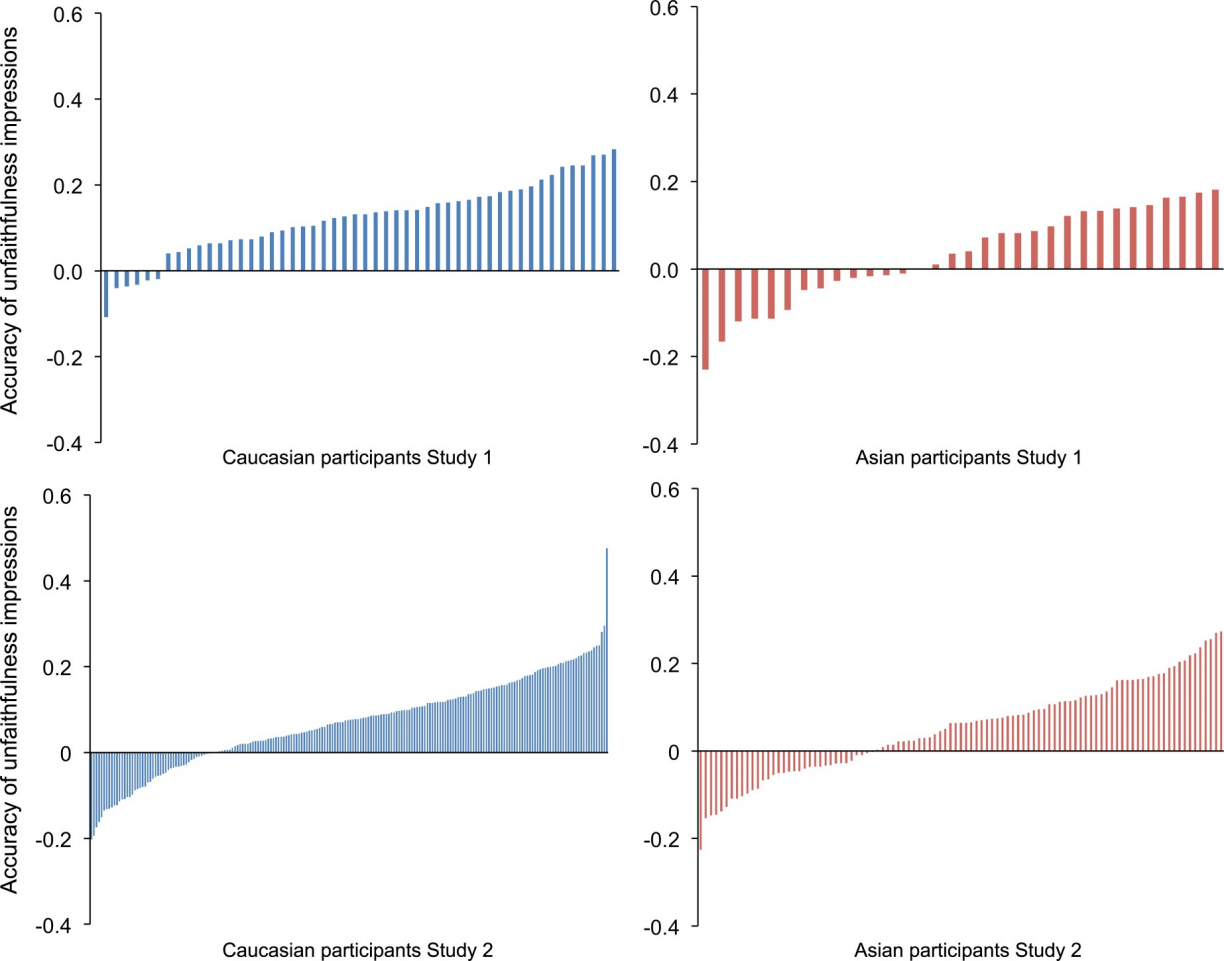
Accuracy of unfaithfulness impressions. Accuracy of unfaithfulness impressions of Caucasian faces for individual Caucasian and Asian participants in Studies 1 and 2. Accuracy is measured as the Pearson’s correlation between each participant’s individual unfaithfulness impressions and actual unfaithfulness (the infidelity index).

We assessed individual accuracy of sexual unfaithfulness judgments, by correlating each individual’s ratings with the infidelity index and comparing these (Fisher corrected) correlations against zero. The Caucasian women showed significant accuracy at judging sexual unfaithfulness at the individual level: mean *r* = .12, SD *r* = .09, *t*(49) = 9.64, *p* < .001 (**Fig 1**). However, the Asian women did not: mean *r* = .03, SD *r* = .11, *t*(31) = 1.62, *p* = .12 (**Fig 1**). Again, the Caucasian women were significantly more accurate than the Asian women: *t*(80) = 4.12, *p* < .001, *d* = 0.90. The proportion of individual Caucasian (18%) and Asian women (0%) who showed above-chance accuracy was also significantly different (proportion *Z*-test = 2.54, *p* = .011, *n* = 82). Therefore, results across individuals replicate the group-level results.

#### Facial recognition performance

Finally, we tested facial recognition by entering the participants’ overall scores on the Australian and Chinese CFMT into a two-way mixed ANOVA, with face race (Australian versus Chinese CFMT) as a within-subjects factor and participant race (Caucasian versus Asian) as a between-subjects factor. One Asian participant did not complete the Chinese CFMT due to computer error. There was a significant interaction between participant and face race: *F*(1,79) = 51.44, *p* < .001, η_p_^2^ = .39, reflecting the expected other-race effect for facial recognition for both groups. The Caucasian women were significantly more accurate on the Australian CFMT (M = 0.79, SD = 0.08) than the Chinese CFMT (M = 0.76, SD = 0.10), *t*(49) = 2.15, *p* = .036, *d* = 0.33. Importantly, the Asian women were significantly more accurate on the Chinese CFMT (M = 0.83, SD = 0.11) than the Australian CFMT (M = 0.72, SD *=* 0.11), *t*(30) = 8.29, *p* < .001, *d* = 1.00.

## Study 2

In Study 1, the Asian participants had less experience with Caucasian faces than the Caucasian participants, and both groups demonstrated the expected other-race effect in facial identity recognition. Whereas the Caucasian women showed a small degree of accuracy in their unfaithfulness judgements of Caucasian male faces, there was no evidence for any accuracy in the Asian women’s unfaithfulness judgements of the same faces, and thus no support for universality in accuracy of these impressions. We did, however, find high overall agreement between the impressions of Asian and Caucasian women at the group level, consistent with a degree of universality in the impressions themselves. Even so, agreement between Caucasian own-race perceivers was higher than between Asian perceivers for attractiveness and masculinity, suggestive of a more general, subtle own-race bias in facial impression formation.

One potential explanation of our Study 1 findings is that accurate judgement of sexual unfaithfulness is not a universal ability. However, the Asian women in Study 1 had reduced experience with Caucasian faces. Thus, their lack of accuracy could simply result from a strong other-race effect [36,37] combined with already small accuracy for the Caucasian participants. If so, then we would expect Asian women’s facial impressions to be accurate when they had more experience with Caucasian faces.

We tested this idea in Study 2 by recruiting new groups of Asian and Caucasian women who had all spent the majority of their lives in the West (the US or Canada). If accuracy in impressions of sexual unfaithfulness is universal, but requires relevant perceptual experience, then these Asian women should show above-chance accuracy. Moreover, there should be little or no difference between the performance of the Asian and Caucasian women. We expected that Caucasian women would also show above-chance accuracy, replicating Study 1. We also expected to replicate the cross-cultural agreement in unfaithfulness impressions themselves.

### Method

#### Participants

Participants were recruited online via Amazon’s Mechanical Turk. Participants provided written informed consent and ethical approval was provided by the Human Research Ethics Board of the University of Western Australia. We excluded 64 Caucasians and 42 Asians who dropped out, 25 Caucasians and 185 Asians who did not fit the self-reported ethnicity criteria, 4 Caucasians and 12 Asians who took part outside of North America, 7 Caucasians and 2 Asians who were homosexual, 19 Caucasians and 11 Asians who had a duplicate IP address and 6 Caucasians and 5 Asians who were not paying attention (pressing the same button repeatedly, contradictory demographic responses, or who told us not to use their data). Exclusions were decided based on *apriori* criteria and before any analysis.

Our final sample consisted of 202 Caucasian (mean age: 40.3; SD: 12.0; mean years in US/Canada: 39.7, SD: 12.4) and 101 Asian women (mean age: 29.6 years; SD: 10.4; mean years in US/Canada: 24.2, SD: 9.5). The final Asian sample was around a decade younger than the Caucasian sample on average, and unlike Study 1, it proved impossible to age-match the two samples. However, age did not significantly correlate with unfaithfulness accuracy for either the Caucasian (*r* = .067, *p* = .342, *N* = 202) or Asian group (*r* = .194, *p* = .052, *N* = 101), so we retained the full sample in our analyses.

Asian women were Chinese (*n* = 29), Korean (*n* = 24), Japanese (*n* = 13), Taiwanese (*n* = 10), or from Vietnam, the Philippines, Cambodia, or Thailand (*n* = 25). Caucasian women were from the US (*n* = 197), Canada or Western Europe (*n* = 5). As expected, Caucasian women reported significantly more social contact with own-race (M = 5.1, SD = 0.7) than other-race (M = 2.5, SD *=* 1.0) individuals: *t*(201) = 25.84, *p* < .001, *d* = 3.01. However, Asian women did not (own-race M = 4.2, SD = 0.9, other-race M = 4.3, SD = 1.0), *t*(100) = 1.01, *p* = .313, *d* = 0.11.

#### Stimuli and procedure

Stimuli and procedure were identical to Study 1, except that we did not include the CFMT due to time constraints. Participants saw the same set of 100 faces as Study 1. All participants first rated the faces on how likely they were to be unfaithful; then participants rated the same faces for attractiveness (67 Caucasians, 38 Asians), masculinity (75 Caucasians, 31 Asians), or untrustworthiness (60 Caucasians, 32 Asians). Participants then reported their demographic details and completed the contact questionnaire. Finally, participants completed a socio-sexual orientation questionnaire (part of a different study; not analysed here).

### Results and discussion

There was high reliability at the group level for judgments of unfaithfulness (Cronbach’s alpha for Caucasians: 0.95; Asian: 0.87), attractiveness (Caucasians: 0.96; Asian: 0.91), and masculinity (Caucasians: 0.96; Asian: 0.91), and reasonable reliability for untrustworthiness (Caucasians: 0.81, Asian: 0.52). We averaged across raters to obtain a mean rating for each face on each attribute for Caucasian and Asian perceiver groups separately (**Table 3**). As before, due to non-normality of the infidelity index, we used Kendall’s Tau as well as parametric correlations. Caucasian and Asian attractiveness ratings and Caucasian untrustworthiness ratings were also non-normal (Kolmogorov-Smirnov tests < .113, *p* < .05; see **Table 3** for skew and kurtosis). As for Study 1, we additionally examined the cheating data using negative binomial models to ascertain the robustness of the conclusions (**S1 Text**). These provided identical conclusions as the analyses reported in the main text.

**Table 3:** Descriptive statistics for social judgments of male Caucasian faces by Caucasian (*N* = 202) and Asian women (*N* = 101), Study 2.

		Mean	SD	Range	Skew	Kurtosis
Caucasian impressions	Unfaithfulness	5.4	0.6	3.8–7.0	-0.10	-0.21
	Attractiveness	3.9	1.0	2.0–6.9	0.50	0.07
	Masculinity	6.6	0.9	4.6–8.6	-0.26	-0.46
	Untrustworthiness	5.4	0.6	4.1–7.7	0.74	1.23
Asian impressions	Unfaithfulness	5.7	0.5	4.4–7.1	-0.07	0.78
	Attractiveness	3.3	0.8	1.9–6.2	1.03	1.54
	Masculinity	6.4	0.8	4.2–8.0	-0.43	-0.32
	Untrustworthiness	5.8	0.5	4.8–6.8	0.17	-0.36

#### High cross-cultural impression agreement

All impressions showed strong agreement between the groups, demonstrating considerable cross-race similarity (all *r* >.77, all *p* < .001; **Table 4**). Although the Caucasian women rated the faces as significantly less likely to be unfaithful: *t*(301) = 2.31, *p* = .021, *d* = 0.25, and significantly more attractive: *t*(103) *=* 2.17, *p* = .032, *d* = 0.46, than the Asian women, these mean differences were slight: both unfaithfulness and attractiveness *d* < 0.35; no other significant differences: *t* < 1.48, *p* > .143, *d* < .35 (**Table 3**). The impression variances were not significantly different across the groups (Levene’s tests all *F* < 1.83, all *p* > .179; **Table 3**). Overall, very similar impressions were formed by Asian and Caucasian women, replicating Study 1, and consistent with the idea of universality. We also computed two-way random single measures ICCs to measure agreement amongst individual raters: unfaithfulness (Caucasian .09, CI = .07-.12, Asian .06, CI = .05-.08), attractiveness (Caucasian .28, CI = .23-.35, Asian .21, CI = .17-.27), masculinity (Caucasian .24, CI = .19-.30, Asian: .24, CI = .19-.30) and untrustworthiness (Caucasian .07 CI = .05-.08, Asian: .03, CI = .02-.05). Agreement in judgments was significantly lower for Asian than Caucasian raters for untrustworthiness only, based on comparison of the confidence intervals [31].

**Table 4:** Relationships amongst impressions of Caucasian male faces made by Caucasian and Asian women in Study 2, and between these impressions and actual infidelity (infidelity index). Relationships measured by Kendall’s tau (above diagonal) and Pearson’s *r* (below diagonal). *P*-values shown underneath.

	Infide. index	Caucasian impressions				Asian impressions			
		Unfaith.	Attract.	Masc.	Untrust.	Unfaith.	Attract.	Masc.	Untrust.
**Infidelity index**		.13	-.01	.15*	.11	.11	-.02	.14	.13
		.09	.905	.049	.126	.124	.804	.062	.089
**Caucasian impressions**									
Unfaithfulness	.23*		.26**	.55**	.44**	.66**	.16*	.51**	.38**
	.019		< .001	< .001	< .001	< .001	.017	< .001	< .001
Attractiveness	-.01	.41**		.20**	-.18**	.11	.71**	.21**	-.23**
	.919	< .001		.003	.009	.108	< .001	.002	.001
Masculinity	.23*	.76**	.33**		.40**	.46**	.06	.80**	.34**
	.022	< .001	.001		< .001	< .001	.367	< .001	< .001
Untrustworth.	.26**	.61**	-.28**	.53**		.50**	-.24**	.38**	.58**
	.009	< .001	.005	< .001		< .001	< .001	< .001	< .001
**Asian impressions**									
Unfaithfulness	.20*	.86**	.17	.66**	.69**		.02	.44**	.43**
	.048	< .001	.089	< .001	< .001		.814	< .001	< .001
Attractiveness	-.01	.29**	.89**	.15	-.35**	.05		.07	-.29**
	.935	.004	< .001	.128	< .001	.593		.276	< .001
Masculinity	.20*	.72**	.34**	.94**	.53**	.64**	.14		.31**
	.049	< .001	.001	< .001	< .001	< .001	.169		< .001
Untrustworth.	.16	.51**	-.33**	.47**	.77**	.59**	-.37**	.45**	
	.107	< .001	.001	< .001	< .001	< .001	< .001	< .001	

** *p* < .01

*p < .05, all *N* = 100.

#### Accuracy in unfaithfulness impressions for both Asian and Caucasian women

We assessed accuracy in impressions of unfaithfulness by correlating the average unfaithfulness ratings with the infidelity index. As in Study 1, Caucasian impressions showed above-chance accuracy: *r* = .24, *p* = .019, *N* = 100, 95% CI: .04 to .40 (**Table 4**; the non-parametric correlation was marginally significant: tau = .13, *p* = .090). Critically, Asian impressions also showed above-chance accuracy: *r* = .20, *p* = .048, *N* = 100, 95% CI: .004 to .38 (**Table 4;** the non-parametric correlation was not significant: tau = .11, *p* = .124). The difference between the Caucasian and Asian women was not significant: *Z* = 0.713, *p* = .476, *N* = 100 (*Z*–test from [32]). Thus, impressions of unfaithfulness for other-race faces can have a kernel of truth, given appropriate experience with that population.

#### Facial cues that mediate accurate unfaithfulness impressions

For both Caucasian and Asian women, masculinity ratings correlated significantly with both unfaithfulness ratings and the infidelity index **(Table 4**), making masculinity a potential mediator of accuracy, as in Study 1. This mediation was confirmed, with partial correlation between unfaithfulness ratings and the infidelity index becoming non-significant when masculinity was controlled, for Caucasian (partial *r* = .097, *p* = .338, *N* = 100) and Asian (partial *r* = .096, *p* = .346, *N* = 100) women.

Unexpectedly, untrustworthiness ratings also correlated significantly with the infidelity index for Caucasian participants, at least given parametric correlations (**Table 4).** Moreover, untrustworthiness also significantly mediated the Caucasian unfaithfulness accuracy (partial *r* = .099, *p* = .330, *N* = 100). Therefore, the unfaithfulness impressions cannot be clearly dissociated from more general impressions of untrustworthiness here, but we note that this was not the case in Study 1 or in previous research [10]. Attractiveness was not a mediator as it was unrelated to infidelity for both Caucasian and Asian participants (**Table 4**).

#### Individual-level accuracy in unfaithfulness impressions

We assessed whether Caucasian and Asian women were individually accurate in their sexual unfaithfulness judgments, by correlating each individual’s ratings with the infidelity index and comparing these (Fisher-transformed) correlations to zero. Critically, both the Caucasian women and the Asian women were significantly accurate at judging sexual unfaithfulness at the individual participant level, although the effect was very small: Caucasian mean *r* = .07, SD *r* = .11, *t*(201) = 9.12, *p* < .001; Asian mean *r* = .05, SD *r* = .11, *t*(100) = 4.89, *p* < .001. The Caucasian women were not significantly more accurate than the Asian women at the individual level: *t*(301) = 1.27, *p* = .206, *d* = 0.18, replicating the group level results (**Fig 1**). One Caucasian woman showed unusually high accuracy (*r* = .48, *p* < .001; **Fig 1**); however, results were identical with this participant excluded (Caucasian group accuracy *r* = .229, SD *r* = .10, *p* = .022; average Caucasian individual *r* = .07, one sample t-test *p* < .001). Finally, the proportion of individual Caucasian (13%) and Asian women (9%) who showed above-chance accuracy was not significantly different (proportion *Z*-test = 1.02, *p* = .31, *n* = 303). Thus, the individual-level results replicate the group-level results.

## General discussion

Our studies address a timely and theoretically important question, as to the extent to which facial impressions are universally judged [3,11,23]. Across two studies, we found considerable cross-cultural agreement on impressions of sexual unfaithfulness, with both Asian and Caucasian women forming similar impressions from Caucasian male faces. The finding of strong cross-cultural agreement is consistent with universality in the impressions themselves. Regarding accuracy, across both studies, we found that facial impressions of unfaithfulness of Caucasian men showed above-chance accuracy for Caucasian women [9,10]. In Study 1, Asian women who had spent less than a year in the West were significantly less accurate than Caucasian women, and showed no significant accuracy. In Study 2, Asian women who had spent the majority of their lives in the West showed above-chance accuracy, comparable to that of the Caucasian women. These results support a degree of universality in accuracy of unfaithfulness impressions, given sufficient experience with the race of faces being judged.

Our finding of high cross-cultural agreement in the impressions themselves aligns with a body of work that has repeatedly found cross-cultural consistency for many different facial impressions [6,7,38]. For example, judges in the US agree with judges from the Tsimane’ people in Bolivia on their facial impressions of interpersonal warmth and other key traits [6]. More recently, high cross-cultural agreement in impressions has been observed even for unconstrained, spontaneous impressions of faces [7]. Our findings extend the literature on cross-cultural impressions to include an important social judgment that is crucial for the optimal selection of romantic partners [8,39].

Set against this overall high cross-cultural agreement, we also found some limited evidence of lower agreement for the Asian women compared to the Caucasian women. Specifically, Asian women showed less within-culture agreement than did the Caucasian women for attractiveness and masculinity in Study 1, and untrustworthiness in Study 2. This pattern is in line with a subtle own-race bias in impression agreement, although the effect was not consistent across studies.

Critically, we also found evidence for a degree of universality for accuracy, as Asian women could detect unfaithfulness from other-race faces at above-chance levels in Study 2. We note, however, that the effect sizes were small, especially at the individual level. Thus, in everyday life, for a given observer in a given situation, sexual unfaithfulness perception should not be taken as reliable [40]. Nevertheless, small effect sizes across an evolutionary timespan, can have important consequences at the level of a species [13]. In terms of testing theory, this other-race accuracy is consistent with the adaptive importance of these judgments. In particular, accurate assessments of sexual unfaithfulness could potentially reduce fitness costs associated with unfaithful partners, resulting in selection pressure for accuracy in unfaithfulness impressions across cultural contexts. Indeed, there are also good theoretical reasons to expect such accuracy would be very low, because targets may also be under selection pressure to mask any propensity to be unfaithful [14]. Sexual orientation judgments, also highly relevant for mate choice, have similarly been shown to have modest accuracy across face and perceiver race [11].

Importantly, accuracy depended on experience; that is, we found accuracy for Asian women only when they had grown up in the West and reported similar levels of social contact with Caucasian and Asian individuals. One potential explanation of this finding is that Asian women raised in the West may have more exposure to infidelity than the Asian women raised in the East. However, this account is unlikely, as sexual unfaithfulness is as prevalent, and as stigmatized, in the East as in the West [41,42]. More likely, under a perceptual expertise account, inexperienced perceivers may rely on cues that are only valid in own-race faces, or may struggle to recognize the same valid cues in other-race faces [16]. Alternatively, lower motivation to individuate faces with whom participants had less social contact [15], may also account for the accuracy difference for Asian women across studies. Asian perceivers who spent most of their lives in the East may have paid less attention to the other-race faces, perhaps after failing to perceive them as likely sexual partners, and thereby missed valid unfaithfulness cues. Future research may wish to further test perceptual expertise and social motivation accounts.

In Study 1, only the Caucasian women showed above-chance accuracy in unfaithfulness judgments, likely due to their greater reliance on the valid cue of masculinity than the Asian women. This own-race advantage existed despite considerable cross-cultural agreement on the impressions themselves. This result aligns with the finding that facial judgments of power and political success (hypothetical votes) also show high agreement across perceiver race, but judgments of other-race faces are inaccurate in predicting actual occupational success [22] (but see [43]). Study 1 extends the other-race effect literature [16] by demonstrating a new other-race effect, and supports the recent theory that there might be cross-cultural ‘dialects’ in facial impressions, so that perceivers may be more accurate or more detailed in their impressions of own-race faces [7].

Not all facial judgments show an own-race effect. For example, Rule and colleagues have shown that perceivers from different countries can modestly discriminate sexual orientation for both own- and other-race faces, with no other-race effect [11]. Rule and colleagues [11] examined impressions in a context that may accentuate valid cues (sexual orientation from dating profile photographs), whereas we studied a highly proscribed behavior that is unlikely to be deliberately advertised. Potentially, cross-race accuracy may be more easily disrupted as valid cues become more subtle. It is an interesting question whether an other-race effect can be found for accuracy in other functionally important facial impressions.

### Limitations and future directions

Our study had potential limitations. First, we used self-reported sexual unfaithfulness, thus risking under-reporting of infidelity. However, reporting conditions were carefully designed to encourage honesty; for example, participants returned their answers anonymously by submitting them to a locked box. Moreover, any under-reporting of infidelity would, if anything, lead our results to underestimate accuracy.

Second, our study was limited by the lack of an Asian face database with associated infidelity information, which precluded a fully crossed design. Drawing conclusions from partial designs is problematic when one perceiver group views multiple sets of stimuli, because an observed other-race effect could reflect stimuli sampling differences. Our partial design is not subject to this problem, because both perceiver groups saw the same faces. But could the inaccuracy of Asian women in Study 1 reflect a general inability to accurately judge unfaithfulness from men’s faces, perhaps due to reduced *ancestral* risk of unfaithfulness? Consistent with this idea, testis size, a reliable indicator of ancestral sperm competition across animal taxa [44], is smaller for Asian than Caucasian men [45]. However, this account is ruled out by the results of Study 2, where Asian and Caucasian women showed comparable accuracy. There are also theoretical arguments against this alternative hypothesis: sexual unfaithfulness represents a fitness cost in evolutionary terms across culture, and infidelity shows comparable modern rates between Western and East Asian countries [42], so that Asian women are as likely to have developed sensitivity to sexual unfaithfulness cues for own-race faces. Future research would benefit from large-scale databases that include faces from multiple ethnic backgrounds along with self-reported behavior, as the field turns to address the important question of the universality of facial impressions.

### Conclusions

Across two studies, we found considerable cross-cultural agreement on impressions of unfaithfulness, with Asian and Caucasian women forming similar impressions from the faces of Caucasian men. We also found a small degree of accuracy in Caucasian women’s impressions of sexual unfaithfulness for own-race male faces, replicating previous work [9,10]. Critically, we also found a small degree of accuracy in Asian women’s impressions for the same faces, but only if they had extensive experience with Caucasian faces, due to lengthy residence in the West. These results suggest a degree of universality in the accuracy of facial impressions, provided that perceivers have experience with the faces being assessed.

## Supporting information

S1 Text Negative binomial analyses. (DOCX)

S1 Data Study 1 data. (ZIP)

S2 Data Study 2 Asian data. (ZIP)

S3 Data Study 2 Caucasian data. (ZIP)
