# Supplementary material for: Impressions of sexual unfaithfulness and their accuracy show a degree of universality
Source: PLoS One. 2018 Oct 25;13(10):e0205716. doi: 10.1371/journal.pone.0205716 (PMC6201884; doi:10.1371/journal.pone.0205716)
Supplement: S1 Text — (DOCX) [file pone.0205716.s001.docx]

S1 text: Negative binomial analyses

Study 1

In the overall paper, we analysed the infidelity index to be consistent with previous studies^1^, because our main aim was to compare performance of our Asian sample with that of the Western samples used in those studies. The infidelity index is composed of incidents of both cheating and poaching (see main text for details). However, when people think of sexual unfaithfulness, they may think specifically about cheating rather than poaching. For this reason, we also examined the cheating data separately. Given that this dependent variable represents count data (i.e. instances of cheating), we ran generalised linear models with a negative binomial distribution^2^ (using glmmTMB package^3^ in R version 3.4.) to predict cheating from average sexual unfaithfulness ratings from Asian and Caucasian groups of participants. Mixed effects models were necessary to account for non-independence of the data, as Asian and Caucasian groups both rated the same faces.

Unfortunately, the overall model (see R code below) failed to converge, so we could not directly compare the Asian and Caucasian rater groups. Simpler separate models for the Asian and Caucasian raters did converge, however. The average Caucasian sexual unfaithfulness ratings significantly predicted the cheating data (Caucasian model estimate = 0.78, SE = 0.27, *Z* = 2.88, *p* = .004, N = 100) but the average Asian ratings did not (Asian model estimate = -0.11, SE = 0.48, *Z* = 0.22, *p* = .825, N = 100). Thus, the conclusions from the binomial model analyses agree with the analyses reported in the main text.

Study 2

As in Study 1, we ran generalised linear mixed effects models to predict cheating from average sexual unfaithfulness ratings from Asian and Caucasian groups of participants. Unfortunately, the overall model (see R code below) failed to converge, so we could not directly compare the Asian and Caucasian rater groups. Simpler separate models for the Asian and Caucasian raters did converge, however. The average Caucasian sexual unfaithfulness ratings significantly predicted the cheating data (Caucasian model estimate = 1.02, SE = 0.42, *Z* = 2.44, *p* = .015, N = 100), as did the average Asian ratings (Asian model estimate = 1.10, SE = 0.52, *Z* = 2.11, *p* = .035, N = 100). Thus, the conclusions from the binomial model analyses agree with the analyses reported in the main text.

R code

Overall model (Study 2 ran identical models)

Study1 <- glmmTMB(Cheat ~ CaucasianAsian*unfaith_average + (1| modelid), family="nbinom2", data=dataStudy1Long)

summary(Study1)

Caucasian model

Study1Caucasian<- glmmTMB(Cheat ~ unfaith_average, family="nbinom2", data=dataStudy1LongCaucasian)

summary(Study1Caucasian)

Asian model

Study1Asian<- glmmTMB(Cheat ~ unfaith_average, family="nbinom2", data=dataStudy1LongAsian)

summary(Study1Asian)

References

1. Rhodes, G., Morley, G. & Simmons, L. W. Women can judge sexual unfaithfulness from unfamiliar men’s faces. *Biol. Lett.* **9,** 20120908 (2013).

2. O’hara, R. B. & Kotze, D. J. Do not log-transform count data. *Methods Ecol. Evol.* **1,** 118–122 (2010).

3. Magnusson, A. *et al.* glmmTMB: Generalized Linear Mixed Models using Template Model Builder. R package version 0.1.3. (2017).
